# Warming and temperature variability determine the performance of two invertebrate predators

**DOI:** 10.1038/s41598-020-63679-0

**Published:** 2020-04-22

**Authors:** Sonia C. Morón Lugo, Moritz Baumeister, Ola Mohamed Nour, Fabian Wolf, Meike Stumpp, Christian Pansch

**Affiliations:** 1 Department of Marine Ecology, GEOMAR Helmholtz Centre for Ocean Research Kiel, 24105 Kiel, Germany; 2 Department des Sciences Fondamentales, Universite du Quebec a Chicoutimi 555, Chicoutimi, Quebec G7H 2B 1 Canada; 3 Institute for Chemistry and Biology of the Marine Environment (ICBM), University of Oldenburg, 26129 Oldenburg, Germany; 4 Department of Biology and Geology, Faculty of Education, Alexandria University, 21526 Alexandria, Egypt; 5 Comparative Immunobiology, Zoological Institute, Christian-Albrechts-University Kiel, 24118 Kiel, Germany; 6 Environmental and Marine Biology, Åbo Akademi University, 20520 Åbo, Finland

**Keywords:** Ecology, Climate-change ecology

## Abstract

In a warming ocean, temperature variability imposes intensified peak stress, but offers periods of stress release. While field observations on organismic responses to heatwaves are emerging, experimental evidence is rare and almost lacking for shorter-scale environmental variability. For two major invertebrate predators, we simulated sinusoidal temperature variability (±3 °C) around todays’ warm summer temperatures and around a future warming scenario (+4 °C) over two months, based on high-resolution 15-year temperature data that allowed implementation of realistic seasonal temperature shifts peaking midpoint. Warming decreased sea stars’ (*Asterias rubens*) energy uptake (*Mytilus edulis* consumption) and overall growth. Variability around the warming scenario imposed additional stress onto *Asterias* leading to an earlier collapse in feeding under sinusoidal fluctuations. High-peak temperatures prevented feeding, which was not compensated during phases of stress release (low-temperature peaks). In contrast, increased temperatures increased feeding on *Mytilus* but not growth rates of the recent invader *Hemigrapsus takanoi*, irrespective of the scale at which temperature variability was imposed. This study highlights species-specific impacts of warming and identifies temperature variability at the scale of days to weeks/months as important driver of thermal responses. When species’ thermal limits are exceeded, temperature variability represents an additional source of stress as seen from future warming scenarios.

## Introduction

Global climate change is known to have profound impacts on marine ecosystems that range from distributional shifts and changes in species interactions to shifts in ecosystem function and diversity^1–4^. However, current knowledge on the impacts of climate change is based on experiments testing the effects of mean changes of stressors, rather than considering variability around means, or the role of climate extremes^5^.

Environmental variability occurs at a variety of temporal (and spatial scales), from tidal to diurnal, over stochastic weekly to monthly patterns, and seasonal cycles, to large-scale inter-anneal variability. Irrespective of scale, this variability can impact organisms differently than changes in mean temperature (i.e. trends)^5,6^. High peaks of stressful temperatures might be lethal^7,8^, while excursions to below-stressful conditions can allow for stress relaxation^9^. Thus, in a warming ocean with already stressful mean temperature conditions^10^, environmental variability towards lower temperatures might create vital refuge from stress^9,11^. More theoretically, *Jensen's inequality* hypothesis postulates for non-linear functions (bell-shaped thermal performance curves, with an intermediate optimum), any variation around the mean should rise or lower the performance of an organism, when compared to constant environmental conditions^12^.

Coastal waters are amongst the most valuable and productive ecosystems worldwide, providing a wide range of ecosystem services^1,13^. In the context of global climate changes, function and structure of these shallow coastal ecosystems are exposed to profound and fast changes of various drivers such as changes in temperature, salinity, eutrophication status, oxygen saturation and pH^14–16^, as well as their fluctuations^17–19^, often occurring at very short time scales^20^. In the Western Baltic Sea Kiel Fjord, temperature oscillates seasonally with amplitudes of up to 21 °C (winter to summer)^21^. At a shorter time-scale, summer heatwaves can persist for several weeks. At stochastic patterns, temperatures can occasionally also rise by 4 or 7 °C in two or five days, respectively during spring and summer. Following autumn upwelling events temperatures can drop by 7 °C in only one day^21^. Day to night temperature can oscillate by 6 °C in less than 12 hours^21^. This exposes coastal communities to rapid changes in environmental conditions today^22^, with unknown consequences for the future^10^.

Temperature is the main focus of this study because the temperature shifts predicted are substantial and reliable^10^. In addition, temperature is a key abiotic factor for organisms’ physiology, due to its direct effects on biochemical and cellular processes^23^. Temperature shifts can therefore impose severe constraints on the performance of organisms^24–26^, and even slight temperature shifts can have large effects from the cellular to the ecosystem level^25,27–29^. Pejus temperature is the part of a thermal window of a species that ranges between optimal performance and a species’ critical limits, affecting its aerobic scope (i.e. the sum of basal (or standard) and maximum metabolic rates). Between lower and upper pejus temperatures (i.e. lower and upper tolerance limits), basal metabolic rates increase with increasing temperature until maximal levels^30^. Outside this window, negative performance is mainly a result of limited maximum metabolic rate due to limited oxygen capacity^30^, from where any further drop or rise in temperature will cause severe stress for an individual^31^. Beyond critical temperature limits, metabolic depression or anaerobic energy production occurs, with stress protection mechanisms providing some limited plasticity^30^. In marine organisms, temperature has been shown to have profound effects on physiological functions such as oxygen consumption^32–34^, heart rates^35–37^, feeding^38,39^ and activity^40^. Ectotherms, not able to control their body temperature, are most affected by changes in their thermal environmental^41,42^, which will likely be impacted by variability patterns.

**Figure 1:**
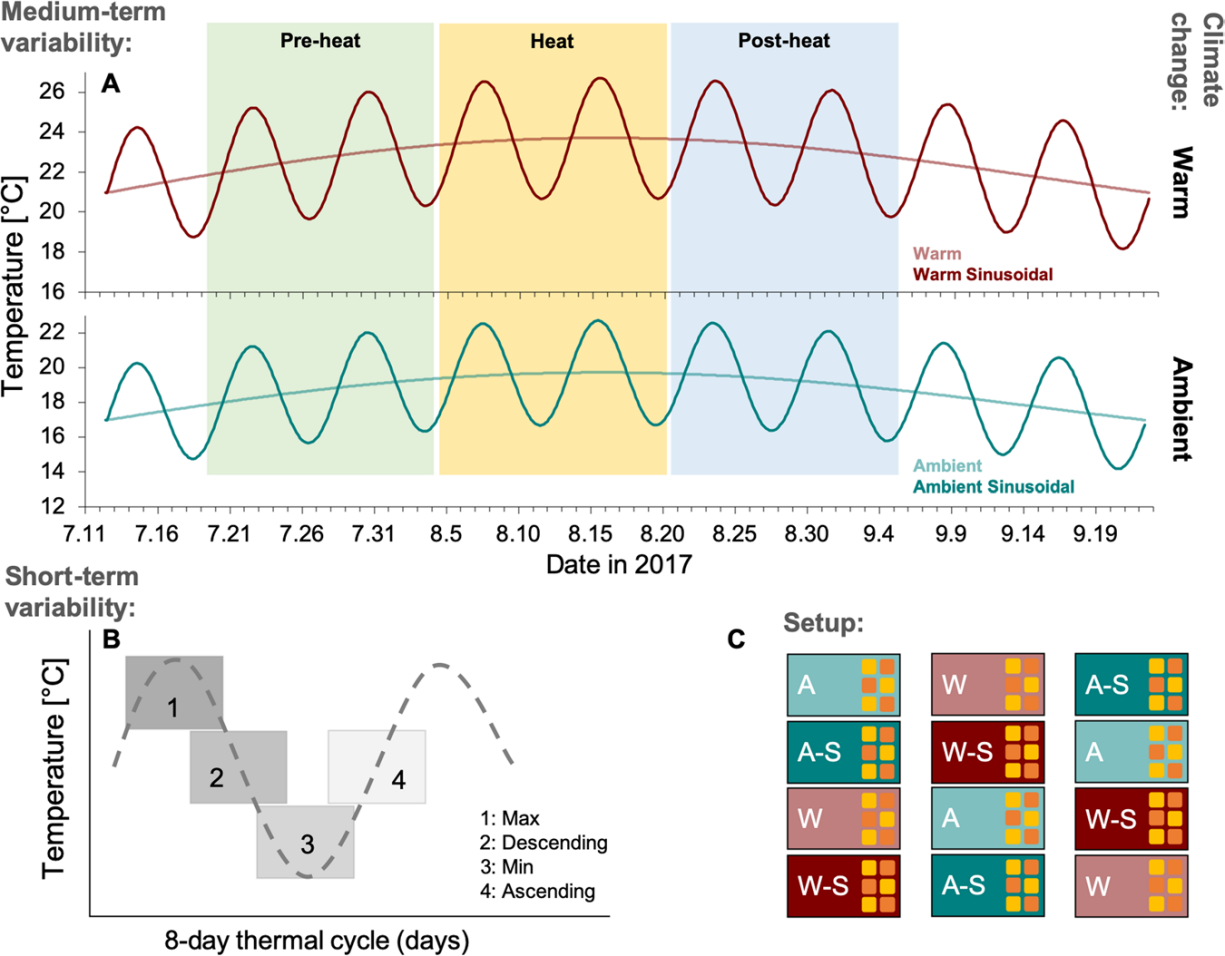
Experimental temperature regimes, experimental design and setup. Implemented temperatures for the four temperature treatments, Ambient (**A**), Ambient Sinusoidal (A-S), Warm (W) and Warm Sinusoidal (W-S; **A**). Energy uptake of individuals was summed at three different scales: (i) at the scale of *short-term* variability (2-day blocks in repeated 8-day sinusoidal temperature cycles, summed over the entire experiment: maximum temperature (1: Max), mean temperature after the maximum (2: Descending), minimum temperature (3: Min) and mean temperature after the minimum (4: Ascending); **B**), (ii) at the *medium-term* variability scale of seasonal summer temperature shifts (energy uptake as sum over 16-day blocks indicated by coloured bars in (**A**): Pre-heat, Heat and Post-heat), as well as (iii) at the scale of *climate change* impacts (identified by comparisons of ambient vs. warm treatments in ‘constant’ and sinusoidal fluctuating settings, using energy uptake as sum and growth over the entire ~2-months experimental duration; compare upper and lower diagrams in **A**). The treatments were realized within twelve indoor mesocosms, three mesocosms serving one temperature treatment (each mesocosm contained six 2 L experimental units, three of which contained single individuals of *Asterias rubens* (orange) and three of which contained single individuals of *Hemigrapsus takanoi* (yellow), N = 9; **C**).

The current study elucidates the role of environmental temperature variability as an amplifier or buffer of mean climate changes in two dominant predator species of the Western Baltic Sea. In an indoor mesocosm system^21^, feeding rates on the blue mussel *Mytilus edulis* were evaluated, for the native common sea star *Asterias rubens* and the invasive brush-clawed shore crab *Hemigrapsus takanoi*. Over two months, both species were exposed to a temperature curve that followed a natural seasonal signal and that peaked midpoint, at ambient (representing todays’ warm summer conditions) and a future warming scenario (+4 °C; Fig. 1A). In addition, sinusoidal temperature cycles (±3 °C, at wavelengths of 8 days) were imposed around these seasonal shifts (Fig. 1A). Growth was assessed from start- and end-weight measurements of individuals (Fig. 1A). Feeding was assessed every other day, converted into energy uptake, and summed for different phases/periods of the experiment. This allowed for assessing the responses of these key predators, at three different scales: (i) to a long-term incubation under a *climate change* trend scenario of +4 °C for ~2 months in ‘constant’ (allowing a seasonal temperature cycle) and fluctuatin g (sinusoidal fluctuations) settings, resembling the current state of climate change experiments (i.e. this scale considered the entire experimental period; Fig. 1A), at a finer scale, to (ii) different phases along *short-term* environmental temperature variability (i.e. sinusoidal cycles over 8 days, leaving 2-day feeding phases, during maximum (Max), Descending, minimum (Min) and Ascending temperature regimes; and at ambient and warm means; Fig. 1B), and to (iii) a *medium-term* (16 days) response scale (i.e. feeding during Pre-heat (before summer peak temperatures), Heat (peak summer temperatures) and Post-heat (following summer peak temperatures) periods; Fig. 1A). The data in the manuscript will therefore be presented and discussed in the following order: long-term climate change scenarios as overall and most relevant response scale (i), short-term sinusoidal variability partly explaining the overall findings (ii), and medium-term shifts (iii), for the two species investigated: *A. rubens* and *H. takanoi*. The experimental units were distributed among 12 mesocosm tanks (Fig. 1C) of the KIBs mesocosm infrastructure^21^.

## Results

### Survival, overall energy uptake and growth of *A. rubens*

**Figure 2:**
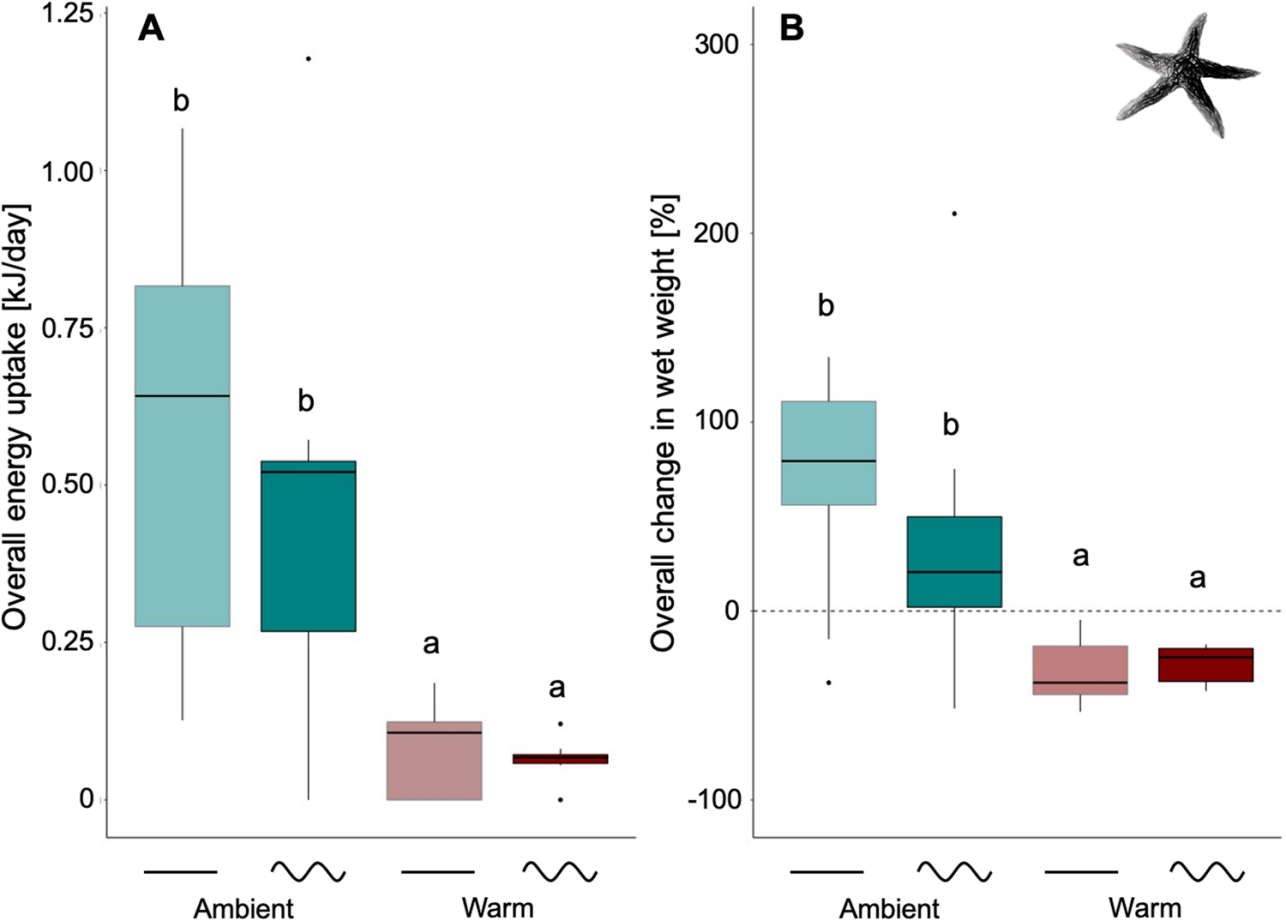
Energy uptake and change in wet weight of *Asterias rubens*. Energy uptake (**A**) and change in wet weight (**B**) during 72 days of experimentation, under ambient or warm and ‘constant’ or sinusoidal fluctuating temperature conditions (sum over the entire period, *climate change* in Fig. 1A). Data are presented as boxplots (median, upper and lower quartile (75^th^ and 25^th^ percentile), whiskers (1.5 times the interquartile range, outliers; N = 9). Significant differences between single treatment combinations were identified using a post hoc Tukey test at p < 0.05 and are indicated by lower-case letters.

All *A. rubens* individuals survived the entire experiment in response to the applied temperature treatments (realized in comparison to anticipated treatments are shown in Fig. S1). Energy uptake (sum of energy consumed over the entire 2-months experimental period; i.e. at the scale of *climate change* in Fig. 1A) of *A. rubens* was significantly affected by mean temperature (GLMER: χ^2^ = 17.53, p < 0.001), being 86% lower (means over ‘constant’ and sinusoidal fluctuating, Table S2) under warm compared to ambient temperature conditions (Tukey test: p < 0.05; Fig. 2A). Although sinusoidal fluctuations (0.45 ± 0.26 kJ/day, mean and 95% CIs; Table S2) reduced mean energy uptake of *A. rubens* by 22% when compared to ‘constant’ conditions (0.58 ± 0.24 kJ/day) in the ambient treatments, no statistically significant effect of fluctuations was detected by the model due to high inter-individual variability (GLMER: χ^2^ = 0.33, p = 0.56; Fig. 2A). No interaction of both factors was observed (GLMER: χ^2^ = 0.0004, p = 0.98; Fig. 2A).

Mean temperature significantly impacted growth of *A. rubens*, measured as change in wet weight (start- vs. end-measurements over the 2-months period; LMER: F = 21.21, p < 0.001). As sea stars even lost substantial weights under heat stress conditions, when compared to ambient temperature conditions (g wet weight change as mean over both, ambient and ambient sinusoidal), biomass in the warm treatments was reduced by 157% (mean over both, warm and warm sinusoidal; Table S2), irrespective of fluctuations (Tukey test: p < 0.05; Fig. 2B). A similar pattern was observed for dry weight at the end of the experiment (GLMER: χ^2^ = 31.6, p < 0.001; Tukey test: p < 0.05; Fig. S2A). Again, although sinusoidal fluctuations (5.17 ± 8.52 g, mean and 95% CIs; Table S2) reduced the overall change in wet weight by 49% when compared to ‘constant’ (10.13 ± 6.69 g) conditions at ambient temperatures, no significant effect of fluctuations was found on the overall change in wet weight due to very high inter-individual variability in responses (LMER: F = 0.33, p = 0.57; Fig. 2B). This was also true for final dry weight of *A. rubens* (GLMER: χ_2_ = 0.50, p = 0.47; Fig. S2A), and no interactions of factors were found for growths (LMER: F = 1.01, p=0.34) or final dry weight (GLMER: χ^2^ = 0.76, p = 0.38).

### Energy uptake of *A. rubens* at the scale of short-term sinusoidal temperature variability

**Figure 3:**
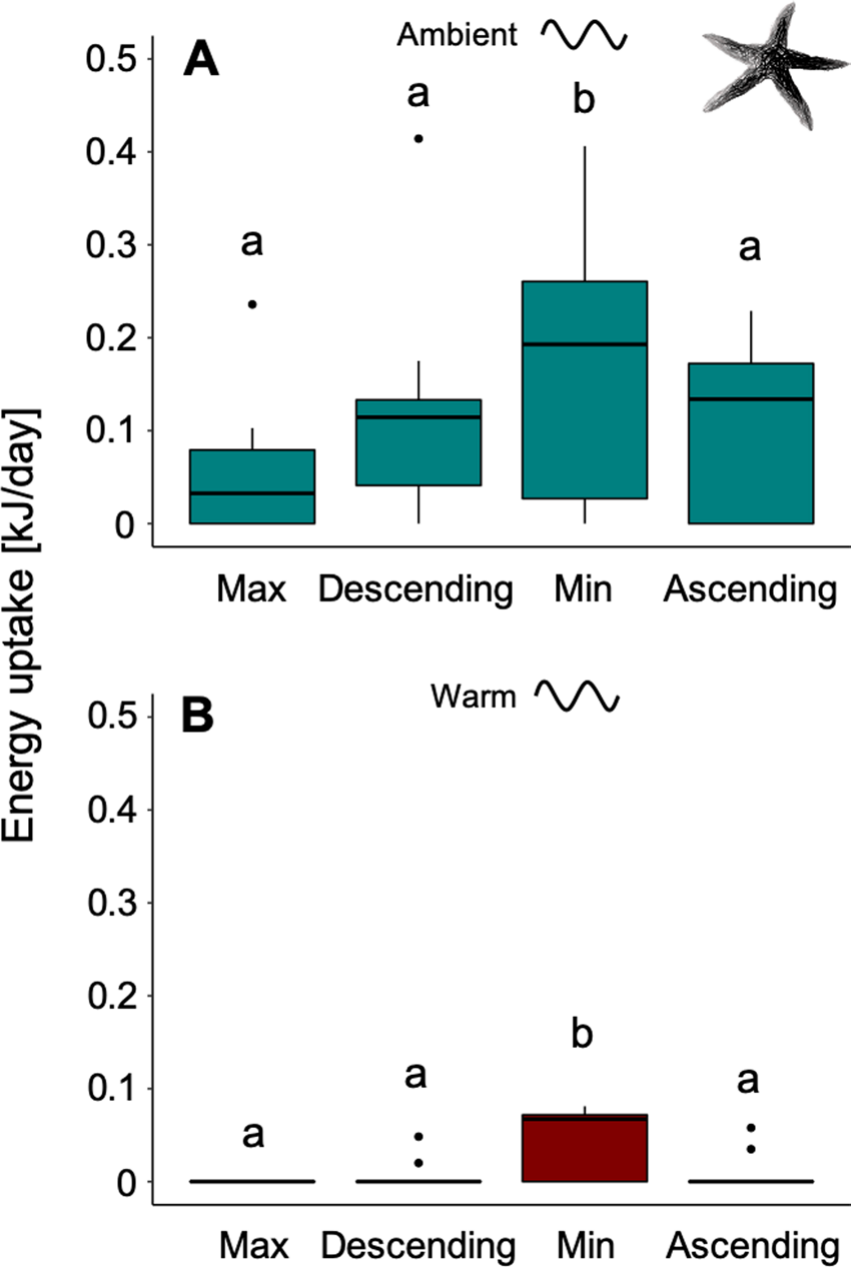
Phase dependent energy uptake of *Asterias rubens*. Energy uptake over 72 days of experimentation, summed for the four phases of the sinusoidal fluctuating cycle (*short-term* variability scale) for the ambient sinusoidal (**A**) and the warm sinusoidal (**B**) treatments, at maximum temperature (Max), mean temperature after the maximum (Descending), minimum temperature (Min) and mean temperature after the minimum (Ascending). All phases of a cycle are also illustrated in Fig. 1B. Data are presented as boxplots (median, upper and lower quartile (75^th^ and 25^th^ percentile), whiskers (1.5 times the interquartile range, outliers; N = 9). Significant differences between single treatment combinations were identified using a PERMANOVA pair-wise test at p < 0.05 and are indicated by lower-case letters.

In order to investigate the *short-term* responses of organisms to temperature variability, energy uptake of 2-day phases (blocks in repeated 8-day sinusoidal temperature cycles: Max, Descending, Min, Ascending; short-term variability scale) was summed over the entire 2-months period (Fig. 1B). Energy uptake of *A. rubens* was significantly affected by mean temperature (PERMANOVA: Pseudo-F = 75.22, p = 0.001) with higher energy uptake under ambient compared to warm temperatures (PERMANOVA pair-wise test: p < 0.05; Fig. 3). Feeding was also significantly different between phases of the sinusoidal temperature fluctuation cycle (PERMANOVA: Pseudo-F = 6.57, p = 0.001), but no significant interaction between mean temperature and phase was detected (PERMANOVA: Pseudo-F = 2.19, p = 0.10; Fig. 3, see also Fig. S3). Individuals fed more during the Min phase when compared to Max, Ascending and Descending, for both mean temperatures (PERMANOVA pair-wise test: p < 0.05; Fig. 3A, see also Fig. S3B). At warm temperatures, no individual sea star ever fed during the Max temperature phase (Fig. 3B, see also Fig. S3D). When feeding was recorded in this generally warm treatment, this had happened partly during mean- (Descending and Ascending) but mostly during minimum (Min) temperature phases (Fig. 3B, see also Fig. S3D).

### Energy uptake of *A. rubens* at the scale of medium-term temperature variability

**Figure 4:**
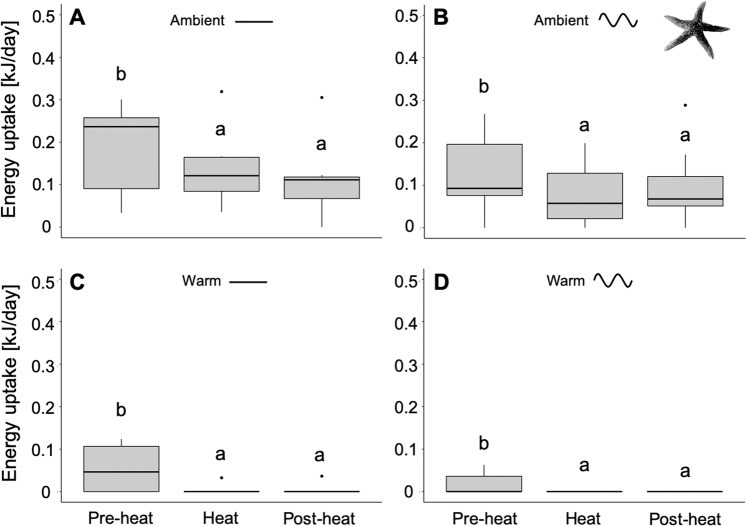
Period dependent energy uptake of *Asterias rubens*. Energy uptake under ambient (**A**), ambient sinusoidal (**B**), warm (**C**) and warm sinusoidal (**D**) temperature treatments (*medium-term* variability scale). Data were separated into three equally long 16-day time periods: Pre-heat (July 19^th^ to August 4^th^), Heat (August 4^th^ to August 20^th^) and Post-heat (August 20^th^ to September 5^th^). Data are presented as boxplots (median, upper and lower quartile (75^th^ and 25^th^ percentile), whiskers (1.5 times the interquartile range, outliers; N = 9). Significant differences between single treatment combinations were identified using a post hoc Tukey test at p < 0.05 and are indicated by lower-case letters.

In order to investigate responses at the scale of *medium-term* variability, energy uptake was summed over 16-day periods during Pre-heat, Heat and Post-heat of the experiment (see medium-term variability scale in Fig. 1A). Energy uptake of *A. rubens* was significantly affected by mean temperatures (PERMANOVA: Pseudo-F = 30.56, p = 0.001) and by heat period (i.e. medium-term variability; PERMANOVA: Pseudo-F = 11.17, p = 0.001). Here, no significant effect was detected from sinusoidal temperature fluctuations (PERMANOVA: Pseudo-F = 1.52; p = 0.262); neither were there significant interactions between mean temperature and heat period (PERMANOVA: Pseudo-*F* = 1.83, p = 0.164), or between sinusoidal temperature fluctuations and heat period (PERMANOVA: Pseudo-F = 0.55, p = 0.576). Individuals showed a higher energy uptake at Pre-heat when compared to Heat and Post-heat, in both ‘constant’ and fluctuating temperature conditions (PERMANOVA pair-wise test: p < 0.05; Fig. 4A,B). Almost no feeding was observed during and after the Heat (peak-summer) period, in ‘constant’ and sinusoidal fluctuating conditions of the warm treatment (Fig. 4C,D). Noteworthy, under warm conditions, feeding stopped 10 days earlier in the sinusoidal fluctuating compared to the ‘constant’ treatment (Fig. S3).

### Survival, overall energy uptake and growth of *H. takanoi*

Three individuals of the crab *H. takanoi* died during the experiment in response to the applied temperature  treatments. One dead individual was found in ambient sinusoidal and two dead individuals were found in warm sinusoidal. Individuals died mostly during moulting. Two individuals moulted without claws (in ambient and warm). These five *H. takanoi* individuals (three dead animals, and two without claws) were removed from all further analyses.

**Figure 5:**
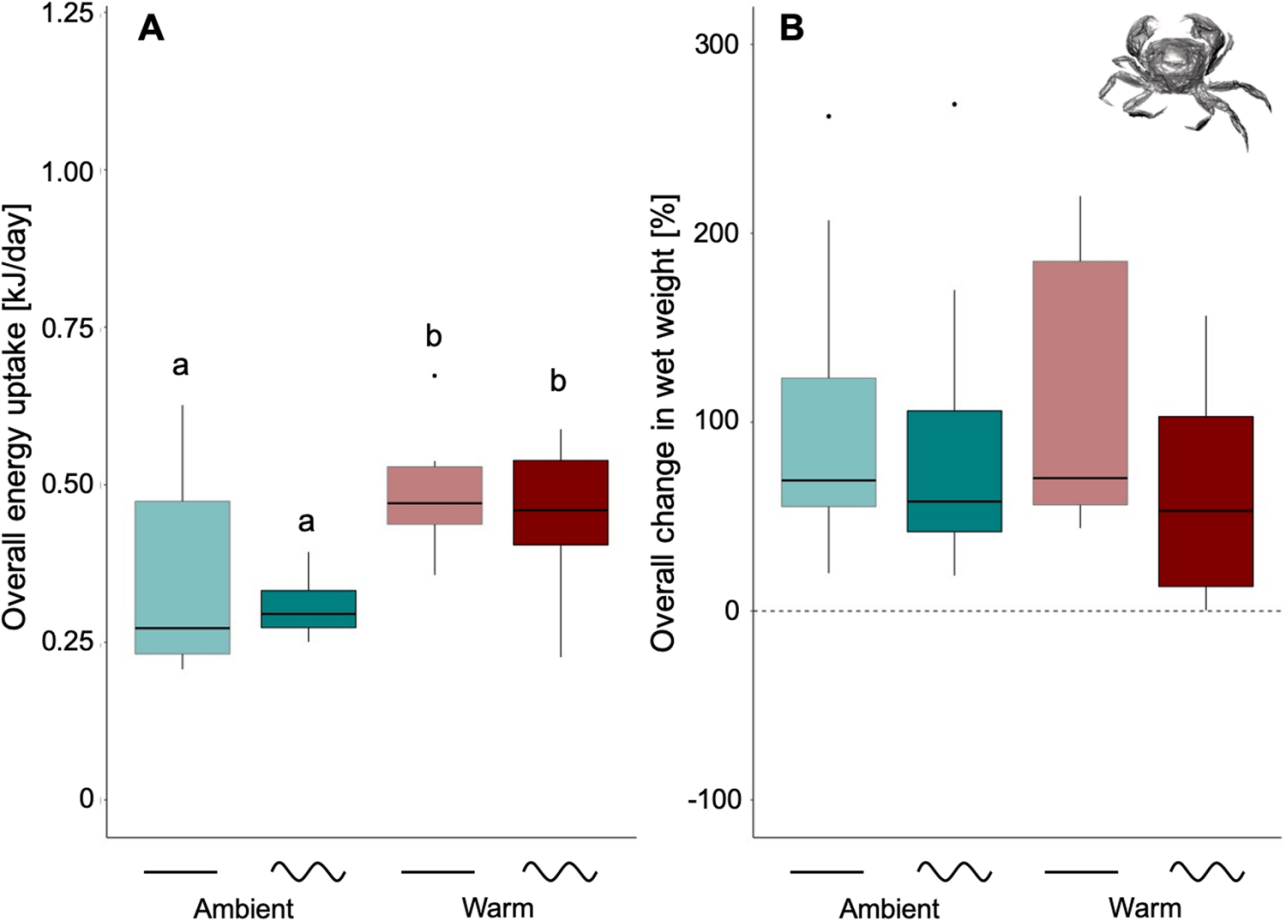
Energy uptake and change in wet weight of *Hemigrapsus takanoi*. Energy uptake (**A**) and change in wet weight (**B**) during 64 days of experimentation, under ambient or wa rm and ‘constant’ or sinusoidal fluctuating temperature conditions (sum over the entire period, *climate change* in Fig. 1B). Data are presented as boxplots (median, upper and lower quartile (75th and 25th percentile), whiskers (1.5 times the interquartile range, outliers; N = 9). Significant differences between single treatment combinations were identified using a post hoc Tukey test at p < 0.05 and are indicated by lower-case letters.

Energy uptake (as sum of energy taken up over the entire 2-months experimental period; i.e. at the scale of *climate change* in Fig. 1A) of *H. takanoi* was significantly affected by mean temperature (LMER: F = 10.78, p < 0.01), being 42% (means over ‘constant’ and sinusoidal fluctuating) higher under warmed compared to ambient conditions (Tukey test: p < 0.05; Fig. 5A). Sinusoidal fluctuations showed no significant effect on the response within the respective temperature treatment (LMER: F = 0.83, p = 0.38; Fig. 5A), and there was no statistically significant interaction between both factors (LMER: F = 0.006, p = 0.94; Fig. 5A). Neither mean temperature (change in wet weight: GLMER: χ_2_ = 0.17, p = 0.67; Fig. 5B; final dry weight: LMER: F = 3.19 p = 0.1; Fig. S2B), nor fluctuations (change in wet weight: GLMER: χ_2_ = 1.11, p = 0.29; Fig. 5B; final dry weight: LMER: F = 0.51, p = 0.48; Fig. S2B), significantly affected change in wet weight or final dry weight of *H. takanoi*, nor were there any interactions between the two factors (change in wet weight: GLMER: χ_2_ = 0.46, p = 0.49; Fig. 5B; final dry weight: LMER: F = 0.15, p = 0.70; Fig. S2B).

### Energy uptake of *H. takanoi* at the scale of short-term sinusoidal temperature variability

**Figure 6:**
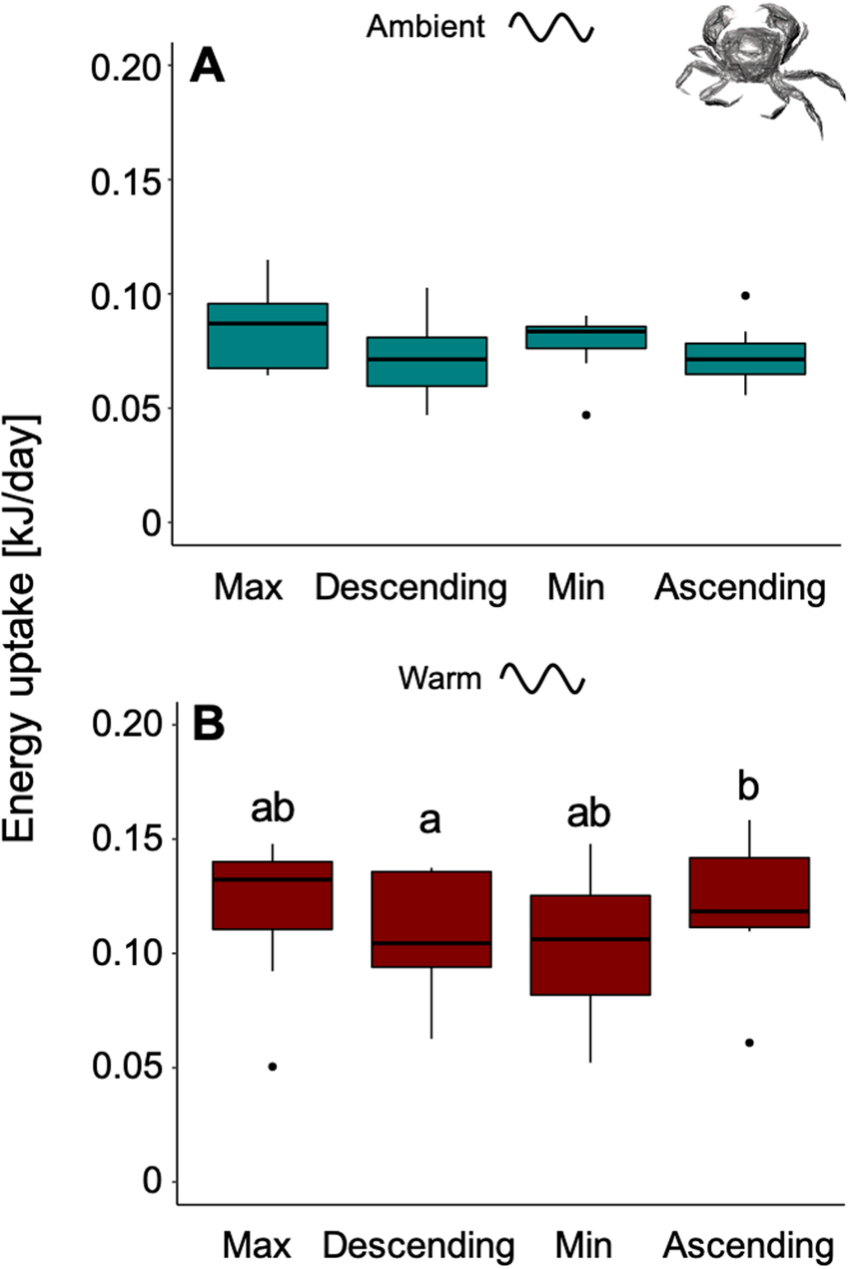
Phase dependent energy uptake of *Hemigrapsus takanoi*. Energy uptake over 64 days of experimentation, summed for the four phases of the sinusoidal fluctuating cycle (*short-term* variability scale) for the ambient sinusoidal (**A**) and the warm sinusoidal (**B**) treatments, at maximum temperature (Max), mean temperature after the maximum (Descending), minimum temperature (Min) and mean temperature after the minimum (Ascending). All phases of a cycle are illustrated in Fig. 1B. Data are presented as boxplots (median, upper and lower quartile (75th and 25th percentile), whiskers (1.5 times the interquartile range, outliers; N = 9). Significant differences between single treatment combinations were identified using a post hoc Tukey test at p < 0.05 and are indicated by lower-case letters.

For energy uptake (summed over the entire 2-months period for the respective *short-term* phases, i.e. 2-day blocks in repeated 8-day sinusoidal temperature cycles (Max, Descending, Min, Ascending) see above and Fig. 1B), mean temperature significantly affected energy uptake of *H. takanoi* (LMER: F = 12.22, p = 0.006; Figs. 6 and S4). Energy uptake also differed significantly between phases of the sinusoidal fluctuation curve (LMER: F = 5.28, p = 0.002; Figs. 6 and S4), but there was no significant interaction between mean temperature and phase (LMER: F = 0.67, p = 0.57; Figs. 6 and S4). Individuals showed a lower energy uptake at the descending phase when compared to the ascending phase (Tukey test: p < 0.05; Fig. 6). Generally, warmer temperatures tended to increase feeding of *H. takanoi* irrespective of the temporal scale in focus (Fig. 6B).

### Energy uptake of *H. takanoi* at the scale of medium-term temperature variability

**Figure 7:**
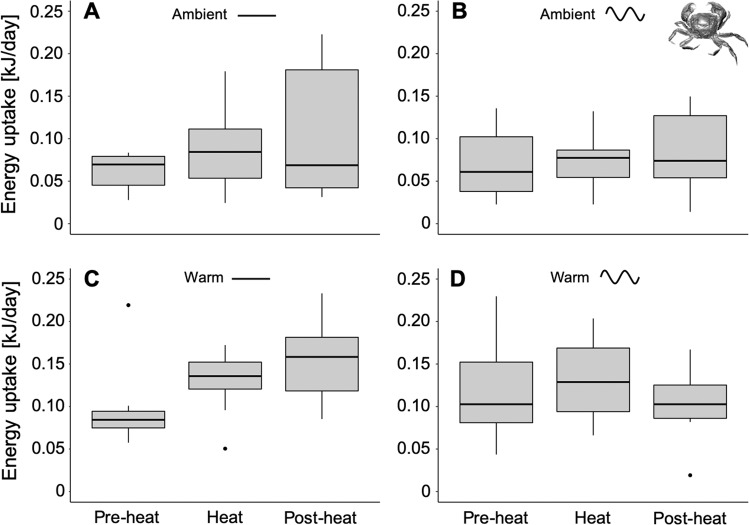
Period dependent energy uptake of *Hemigrapsus takanoi*. Energy uptake under ambient (**A**), ambient sinusoidal (**B**), warm (**C**) and warm sinusoidal (**D**) temperature treatments (*medium-term* variability scale). Data were separated into three equally long 16-day time periods: Pre-heat (July 19th to August 4th), Heat (August 4th to August 20th) and Post-heat (August 20th to September 5th). Data are presented as boxplots (median, upper and lower quartile (75th and 25th percentile), whiskers (1.5 times the interquartile range, outliers; N = 9).

Energy uptake of *H. takanoi* (sum over 16-day periods during Pre-heat, Heat and Post-heat of the experiment, see *medium-term* variability scale in Fig. 1A), was neither influenced by the period of the experiment (LMER: F = 2.42, p = 0.09), nor by sinusoidal fluctuations (LMER: F = 0.64, p = 0.43). Mean temperature, however, affected this trait significantly (LMER: F = 11.10, p = < 0.01) showing higher energy uptake in warmer conditions; Figs. 7 and S4). Generally, *H. takanoi* tended to show a higher energy uptake with experimental time when exposed to ‘constant’ treatments (Fig. 7A,C), while in the fluctuating treatments energy uptake remained rather equal throughout the experiment (Fig. 7B,D; see also Fig. S4).

## Discussion

Warming had a clear negative impact on consumption rates and biomass increase of *A. rubens*. In contrast, warming increased consumption rates of *H. takanoi*, while biomass increase was not significantly differently between treatments. Under the warming scenario, energy uptake of *A. rubens* was only observed at the beginning of the experiment, and thus, *A. rubens* lost significant amount of weight under the warming scenarios. Despite strong sinusoidal temperature fluctuations of 6 °C around the mean, this variability did not significantly impact the final response variables investigated for both species (i.e. summed energy uptake and growth from start to end). However, even though this effect was not statistically significant, *A. rubens* showed lower mean energy uptake as well as reduced growth in sinusoidal fluctuating conditions compared to ‘constant’ conditions, particularly so under ambient temperatures. Generally, inter-individual variability in energy uptake and growth was highest under ambient treatment conditions, but this trait was compressed with increasing stress levels (i.e. ambient> ambient sinusoidal> warm> warm sinusoidal). Interestingly, *A. rubens* suffered earlier and more strongly from heat stress when exposed to temperature variability, likely due to extremes experienced during high peak temperatures. Phases of heat-stress release (low peak phases) allowed for some feeding, and likely recovery, which, however, did not allow for a full compensatory energy uptake. In contrast, energy uptake by *H. takanoi* was highest in the highest phases of the sinusoidal temperature fluctuation cycle. Overall, energy uptake by *H. takanoi* remained rather constant with time (over the two months), while that of *A. rubens* strongly decreased over the experimental period, particularly so under the warming scenarios.

### Warming impacts *A. rubens* and increases energy assimilation of *H. takanoi*

The common sea star *A. rubens* clearly suffered from the warming scenario. This could be due to the high mean temperatures of up to 23 °C imposed by this treatment, which clearly exceeded the thermal tolerance of this species, by strongly decreasing the metabolism (F. Melzner pers. comm.). Based on results found by Aguera *et al*.^43^, the temperature range in which *A. rubens* actively feeds on its prey was shown to be between 3 and 22 °C. Another study on *A. forbesi*, a congener of *A. rubens* from Long Island Sound, USA, showed that feeding rates were strongly impaired by seawater temperatures above 20 °C, leading to weight loss in individuals subjected to temperatures higher than this threshold^44^. Temperatures imposed in the future warming scenarios of the current experimental study mostly exceeded *A. rubens’* thermal tolerance threshold, likely being responsible for the detrimental impacts observed in the warming treatment.

In contrast to the sea star, feeding and growth of the invasive brush-clawed shore crab *H. takanoi* were not negatively impacted by the 4 °C warming scenario. The thermal tolerance of this species is not well described in the literature. Van den Brink *et al*.^45^ show that individuals of this species moult faster and more frequently under increased temperatures (12 °C < 18 °C<24 °C). This indicates that individuals of this species can tolerate temperatures up to 2 4 °C well, and higher, as the data of the current study suggest (with peaks of up to 27 °C). Similar to *H. takanoi*, the congeneric species *H. sanguineus* is very tolerant to a wide range of temperatures^46^, experiencing temperatures from ~0 °C to 28 °C in their native range^47^. Interestingly, however, higher temperature increased feeding rates of *H. takanoi*, likely triggered by increased metabolic rates in warmer seawater conditions. This energy uptake, however, did not translate into energy assimilation, as growth rates were rather similar over all experimental treatments, indicating certain physiological stress to the organisms. To which extend energy conversion was limited in warmed *H. takanoi* individuals, and if this might translate into longer-term constraints, is, however, beyond the scope of this study.

As it is well described in the literature, invasive species tend to be overall better at enduring disturbances than native species^48,49^, possibly explaining parts of the wide temperature tolerance of *H. takanoi* (see also^50,51^). In line with this, recent studies have shown that *H. takanoi* is a successful invader, and a more competitive species than other crabs such as the European shore crab *C. maenas* and the Asian shore crab *H. sanguineus*^45,52^. Overall, this experimental work suggests a wide temperature tolerance of *H. takanoi* and effective compensation mechanism in place that are partly fuelled through increased feeding, suggesting an increasing invasion success in a future ocean of increased mean temperatures and stronger temperature variability.

### Small-scale temperature variability amplifies negative warming impacts in *A. rubens*

For *A. rubens*, for which the thermal tolerance was clearly exceeded in this study, sinusoidal fluctuations in warmed conditions had a stronger impact than warming alone. This might be partly explained by the generally higher temperature peak(s) experienced in the sinusoidal fluctuation treatment (max. 22 °C vs. 27 °C in ‘constant’ compared to ‘sinusoidal fluctuating’ environmental conditions, respectively). Generally, however, *A. rubens* fed more (or exclusively) during minimum temperatures of the sinusoidal fluctuation cycle. This suggests that individuals of *A. rubens* might have taken advantage of these periodically low temperatures, using these as refuge or phases for stress-recovery to gain energy and to overcome the preceding or subsequent heat stress (*sensu* Wahl *et al*.^9^). Organisms can undergo metabolic adjustments in response to fluctuating environmental stimuli^53^. Studies performed by colleagues in the lab (F. Melzner, unpublished data) showed that coelomic fluid *p*O_2_ of *A. rubens* decreases with increasing heat stress. This cellular stress response might reflect the responses of an individual to environmental fluctuations at the tissue or at the organism levels. Overall, however, fluctuations did not help *A. rubens* coping with the high temperature peaks in the long run, which more likely introduced more and accumulative stress that persisted and likely accumulated, preventing recovery. This effect can be observed in the warming scenarios, in which individuals stopped feeding about ten days earlier under sinusoidal fluctuations compared to ‘constant’ conditions. Thus, a positive stress-memory effect (*sensu* Walter *et al*.^54^), i.e. short-term physiological acclimation to stress events, could not be observed for this species. Here, either the single events were too strong or too close to each other, not allowing for metabolic adjustments, a hypothesis that requires future investigations. Therefore, climate change research needs to address whether mean temperature changes or short but extreme events will determine the fate of a species under climate change stress.

For *H. takanoi*, imposed sinusoidal fluctuations around ambient and warmed means showed an opposite pattern. Here, higher temperatures during the sinusoidal fluctuation cycle led to higher feeding rates. This clearly contrasts the prediction that temperature fluctuations amplify the negative impact of future warming stress in ectotherms^55^, and may be explained by a wide thermal tolerance, or by additional energy demands during periods of high temperature stress. Possibly, the applied small-scale temperature variability was likely at a linear section of the thermal performance curve of this species (*sensu* Ruel and Ayers 1999^12^).

### Medium-term temperature variability does not allow for recovery in *A. rubens*

For *A. rubens*, energy uptake did clearly decrease with time, particularly so under the applied warming scenarios. Thus, *A. rubens* was (i) not able to recover from, or adjust to, the peak temperatures applied in the sinusoidal fluctuating treatments as well as from (ii) mean temperatures above 22 °C in the treatment following the seasonal temperature cycle (medium-term temperature variability). This is in line with observations by Melzner *et al*. (F. Melzner, pers. comm.) and Aguera *et al*.^43^ suggesting a critical thermal threshold for this species at around 22 °C. Even under lower temperatures returning towards the end of the experiment, and possibly allowing for recovery, *A. rubens* did not start feeding again, which may be due to the long heat stress period as well as due to the long period of heat-induced starvation, and the therefore accumulated stress^56^. Extended starvation periods (~6 weeks) decrease prey consumption^56^, and can lead to breakdown of pyloric reserves, autolysis and skeleton reabsorption^57^. In the present study, under warming scenarios, individuals of *A. rubens* were starving for more than 4 weeks, before the seasonal signal allowed tolerable temperature levels (<22 °C), likely preventing recovery of *A. rubens* individuals.

### Potential implications at the community level

Considering the importance of predation to shape communities^58^, it is vital to evaluate the interactions between predator species, which share similar prey resources. Clearly, in the Western Baltic Sea, *A. rubens*, *C. maenas* and *H. takanoi* share the same prey source, namely the mussel *M. edulis*. From empirical studies, the common European shore crab *C. maenas* (not part of this study) can tolerate temperatures between 0 °C and 33 °C with optimal temperatures ranging between 10 and 18 °C^59,60^. Other studies showed that the upper thermal tolerance limit for this species varies between 31-35 °C depending on season and acclimation temperatures^61^. Based on this, projected warming of sea surface temperatures towards the end of this century^62^ will likely increase feeding pressure by *C. maenas* and *H. takanoi* on the common prey, *M. edulis*, due to higher metabolic rates and increased nutrition demands by these predators. Thus, the differential impacts of warming in these two crab species compared to the predator *A. rubens* will influence the trophic interactions at the community level.

*A. rubens* can feed on a wide range of mussel sizes (0–50 mm), although it has been observed they prefer medium sizes (15 to 30 mm^63,64^). The maximum *Mytilus* prey size for *A. rubens* from the Baltic Sea was determined to be 48 mm^65^, with larger mussels finding a size refuge from predation^64^. For *H. takanoi*, it is known that this species can open mussels up to 27 mm but the preferred size range is between 10 to 17 mm size^66^. These predators differ in the size of the mussels they prefer to consume, which, in a warming scenario, could cause shifts in the mussel population with increased predatory pressure on smaller-sized mussels from *H. takanoi* and a decreased pressure on larger-sized mussels from *A. rubens*. Accurate ecosystem estimates would, however, need to include the mussels’ responses to the respective scenarios.

## Conclusions

This study demonstrated neutral-positive as well as strongly negative impacts imposed by warming and temperature variability, i.e. by (i) a warmed future ocean scenario, by (ii) medium-scale temperature variability due to seasonal temperature shifts (almost one summer season simulated over ~2 months), as well as (iii) by sinusoidal temperature cycles at the resolution of days. Overall, this study suggests clear species-specific response schemes in reaction to warming and temperature variability, seen in the two investigated species, *A. rubens* and *H. takanoi*, with possible consequences for local ecosystem dynamics. Temperature variability did not allow for recovery during low temperature peaks in *A. rubens*. In contrast, temperature variability increased the stress impact due to extremes, which clearly exceeded *A. rubens’* thermal tolerance. This study highlights impacts at various temporal scales of thermal variability, pleading for more detailed assessments on the impacts from environmental variability in various drivers on species to ecosystem performance, and assessments on how organisms and communities can acclimate and adapt to re-occurring extreme events.

## Methods

### Study system

The Baltic Sea is a relatively species-poor ecosystem, in which small impacts can mean dramatic changes for the subsequent food web^67,68^. This particular sea is strongly impacted by environmental variability, due to a relatively shallow water depth, a high land to water ratio, and the fact of being enclosed with limited excess to full marine seawater conditions^69^. Sporadic inflow events from the North Sea provide high-saline waters to this mostly brackish environment^69^, seasonal pH variability increases in amplitude^70,71^, and wind-driven sporadic up-welling shoals highly acidic and hypoxic water masses^72^. Regular tides are absent, while sporadic wind-driven water level changes occur. All of these factors fluctuate at diverse temporal and spatial scales, from diurnal over stochastic weekly to monthly events, to seasonal and decadal patterns. Within the Kiel Fjord (temperate climatic zone), temperature variability can be immense, from seasonal (>20 °C) to any stochastic and diurnal (6 °C) cycles^7^.

Predators and prey used in this study were the sea star *Asterias rubens* and the crab *Hemigrapsus takanoi*, and their common prey, the mussel *Mytilus edulis*. The bivalve *M. edulis* dominates large parts of Western Baltic Sea benthic communities and provides substrate, habitat and food for other organisms^64^. In the Western Baltic Sea, *A. rubens* is one of the main predators on *M. edulis*, and together with the European shore crab *Carcinus maenas*, controls the abundance of the mussels^64^. The recent invader *H. takanoi* was for the first time recorded in the Western Baltic Sea in 2014, but originates from the Western Pacific^73^. Adults of this predator feed on *M. edulis* (smaller size classes of mussels than adult *A. rubens*^66^, O. Mohamed-Nour, pers. comm.). This species is characterized by rapid growth, a short life cycle and early sexual maturity^74^, thus likely representing a potentially strong competitor to *A. rubens*.

### Species collection

Collection of *A. rubens* took place via snorkelling in a water depth of two to three meter on a sandy substrate on June 26^th^ and June 27^th^ 2017 in the Kiel Fjord, Western Baltic Sea, at Möltenort (54°22.94′N; 10°12.16′E). 36 individuals with a size range of 8 ± 1 cm maximum overall diameter, (24 ± 5 cm^2^ aboral dorsal surface area; 15 ± 5 g wet weight) were used for the experiment. Diameter was measured as the maximum length from arm-tip to the opposite arm-tip, measures with callipers to the nearest mm. Area was measured as the aboral dorsal surface area of the sea star individuals from photographs (taken from above) and ImageJ analyses.

Collection of *H. takanoi* took place in the inner part of the Kiel Fjord at the GEOMAR pier (54°19.7′N; 10°8.95′E), at a sampling depth of two meter. Individuals were collected between June 28^th^ and July 1^st^ 2017 using traps, which consisted of 50 × 50 × 20 cm PVC pipe structures, covered with a 1 mm mesh, with an entrance on one side of the traps were filled with crushed mussels. A total of 36 individuals with a size of 1.4 to 2.4 cm carapace width, measured as the distance between the two middle antero-lateral teeth^75^ and 4 ± 2 g wet weight, were used for the experiment.

After collection, *A. rubens* and *H. takanoi* were acclimated to laboratory conditions in flow-through seawater at 16 °C (±0.2 °C) and the respective field salinity (20.8), at saturated oxygen conditions (achieved by aeration). Therefore, individuals were kept in 600 L tanks of the Kiel Indoor Benthocosms (KIBs^21^) at densities of 36 individuals of each species per mesocosm tank. Individuals were fed mussels *ad libitum*. After 14 days of acclimation, individuals were randomly assigned to the different experimental treatments.

### Experimental setup

The experiments were conducted from July to September 2017 in the Kiel Indoor Benthocosms. The infrastructure consisted of twelve 600 L tanks located in a temperature-controlled room at GEOMAR, Kiel, Germany. Each mesocosm tank was equipped with heaters (Aqua Medic TH 300-500, Bissendorf, Germany) and one cooler (Aqua Medic Titan 2000), all connected to a GHL-Computer (GHL-Profilux 3.1 T, Kaiserslautern, Germany). Pumps (EHEIM, Deizisau, Germany) provided circulation of water through the coolers as well as within the tanks. Header tanks with 60 L each continuously supplied Kiel Fjord seawater to the experimental units. An in-house facility supplied filtered pressurized air.

Over the entire experiment both species were kept in 2 L Kautex bottles (experimental units), at one individual per unit (9 individuals per treatment with a total of 36 individuals per species). Silicon tubes provided aeration and a flow-through of seawater of 300 mL per hour to each unit. Thus, water within each unit was completely renewed every four hours. A total of three units per species were placed into each of the twelve 600 L tanks (Fig. 1C). The target temperature within the units was indirectly adjusted by the surrounding water of the 600 L tank, and was partly preconditioned within the header tanks via passive heat exchangers^21^.

### Temperature treatments

In order to simulate natural background temperatures, a temperature model was applied, based on 15 years (2000 to 2014) of temperature data collected at the GEOMAR pier in 1.5 meter depths (GEOMAR weather station, Ocean Circulation and Climate Dynamics - Marine Meteorology; data provided at Pangaea: 10.1594/PANGAEA.888599). In temperate regions, annual temperature regimes differ strongly between years. Therefore, population resistance will be strongly determined by critically warm temperature peaks occurring in warm years, as critical physiological tolerance limits will be exceeded. Therefore, data from June to September (experimental period) were considered only, from which the eight warmest years (reaching maximum values above 20 °C in summer) were chosen and fitted to a polynomial (4^th^ degree; Fig. S5). This procedure was applied in order to simulate temperature variability in a warm year of today as well as in a warm year projected into the future. This temperature profile represented the ambient non-fluctuating temperature treatment (“ambient”), indicating todays’ average warm summer conditions and including the seasonal signal (Fig. 1A). The “warm” treatment represented the identical polynomial function but with the offset of +4 °C (projected future warming the Baltic Sea^62^). Around these two ‘constant’ but seasonally fluctuating treatments, sinusoidal temperature fluctuations were modelled (“ambient sinusoidal” and “warm sinusoidal”). Nine replicates of one treatment were distributed among three independent mesocosm tanks (see also Fig. 1 for details).

The chosen amplitude and wavelength was 3 °C (i.e. 3 °C above and below mean) and 8 days, respectively. The approach of applying sinusoidal fluctuation patterns does not entirely reflect patterns found in the field^7,21^, but it allows for (1) applying similar means between fluctuating and non-fluctuating treatments as well as (2) similar deviations from the mean between fluctuating treatments. As diurnal temperature patterns may coincide with diurnal natural physiological cycles of organisms, these were (i) intentionally avoided for this approach, and (ii), response variables were always measured at the same time during a day.

### Feeding rate assessments

Both, *A. rubens* and *H. takanoi* were fed *M. edulis* mussels every second day. For each feeding event, freshly collected mussels from the inner part of the Kiel Fjord (54°19.77′N; 10°8.95′E), were provided while old mussels were removed. This way, the mussels had to endure the treatment for a short period of time only, reducing the possibility of treatment effects on mussels and their nutrition, and thus indirectly on the predator species in focus.

Each individual of *A. rubens* received five mussels of 25 to 35 mm size (total length). As for *H. takanoi*, all mussels were consumed during the first two feedings, the number of fed mussels had to be adjusted. While each individual *H. takanoi* received five and 15 mussels on day 2 and day 4, respectively, 20 mussels of 9 to 12 mm were offered from day 6 onwards. Thus, these first two data points (day 2 and day 4) were removed from the dataset. From then, however, at no occasion all mussels were consumed by *A. rubens* or *H. takanoi* before new mussels were added, thus representing *ad libitum* food conditions. Feeding was monitored for a period of 72 and 64 days for *A. rubens* and *H. takanoi*, respectively. For *A. rubens*, the shell of every consumed mussel was measured in order to estimate mussels’ energy content (see details below). In the case of *H. takanoi*, this was not possible, since feeding activity of the crabs destroyed the mussels. Here, the average size (10.5 mm) of the mussels offered was used for estimates on energy uptake.

### Mussel energy content

To calculate energy uptake by *H. takanoi* and *A. rubens*, twelve mussels within the fed size range were frozen at the beginning, twelve in between and twelve at the end of the experiment. The mussel flesh of the 36 mussels was dried (24 hours, 80 °C) in a drying cabinet (MMM Ecocell, Munich, Germany) and weighted (0.0001 g; Sartorius, Berlin, Germany). The shell length of every mussel was measured using a calliper. These data were complemented by an existing dataset from the same laboratory for smaller mussels from the Kiel Fjord^76^; Pangaea dataset: doi:10.1111/gcb.12109). A predictive relationship between soft tissue dry weight of mussels (g) and mussel length (mm) was used (power function with R^2^ = 0.97; Fig. S6). From this, dry mass and energy uptake were calculated according to Brey *et al*.^77^, as 18.85 Joules per mg dry mass of mussels. The respective measure in fed mussel size (*A. rubens*) or an average fed mussel size (*H. takanoi*) were estimated.

### Growth and condition

Biomass (g wet weight) was quantified on July 12^th^ (start) for both species and additionally at the end of the experiment, on September 21^st^ (day 72) for *A. rubens* and on September 14^th^ (day 64) for *H. takanoi*. For this, individuals were weighed on a scale (0.1 g; Kern KB 10000-1 N Balingen-Frommern, Germany), after gently blotting individuals dry with a tissue.

At the end of the experimental period, all individuals of both species were frozen (6 hours at minus 20°C), and dried (48 hours, 80 °C) in a drying cabinet and weighted (0.0001 g; Sartorius, Berlin, Germany). Wet weight was the best predictor of *A. rubens* as well as *H. takanoi* biomass without having to kill the animal (dry weight as assessed at the end of the experiment; Fig. S7), and this measure was therefore used for evaluation of biomass changes over time.

### Identification of responses at different scales

The temperature treatments followed a seasonal pattern, from pre summer benign conditions over summer peak temperatures, to again benign post peak summer conditions (details in Fig. 1). In addition, around these rather ‘constant’ treatments, sinusoidal temperature cycles were simulated (fluctuating treatments; Fig. 1). Growth was assessed from start and end wet-weight measurements as well as from dry weight measurements at the end of the experiment. As feeding was determined every other day, the experiment could be evaluated at distinct scales. For this, energy uptake was summed over the particular scale of interest (see Fig. 1 for details). At the largest scale (*climate change*), the overall sum of energy uptake was calculated for the four respective temperature treatments, indicating ambient vs. warm temperature conditions in ‘constant’ or fluctuating modes. At the smallest scale, representing *small-scale* temperature variability of 8-day wavelength, for 2-day feeding periods at minimum, maximum and mean temperatures after minimum or maximum temperatures during each sinusoidal temperature cycle (Fig. 1B), energy uptake was summed for Min, Max, Descending and Ascending phases. At the *medium scale*, representing longer seasonal periods of 16 days, energy uptake was summed for ‘Pre-heat’ (July 19^th^ to August 4^th^), ‘Heat’ (August 4^th^ to August 20^th^) and ‘Post-heat’ (August 20^th^ to September 5^th^) periods (see Fig. 1 for details).

### Data processing and statistical analysis

In order to evaluate change in wet weight, data were a priori converted to relative growth rates using the formula suggested by Crawley (2012)^78^: weight = log (final weight/initial weight). The purpose of this was to avoid not normally distributed errors associated with percentage/proportional data^78,79^. The data from bi-daily energy up takes were summed over different periods (see Fig. 1 for details): i.e. (1) *short-term* variability (energy uptake during Max, Descending, Min and Ascending of the sinusoidal fluctuation curve) and (2) *medium-term* variability (energy uptake during Pre-heat, Heat and Post-heat).

For this study, a factorial design was applied with two factors: mean temperatures (ambient vs. warm) and fluctuation regime (‘constant’ vs. sinusoidal fluctuating). Analyses and statistical procedures were performed using the statistical software R, version 3.4.0 (http://www.R-project.org) and Primer 7 with PERMANOVA+. Model assumptions, in terms of normality and homogeneity of variances, were evaluated by visual inspection of residuals’ plots, and verified by Shapiro and Levene's tests. Linear Mixed Effect Models (LME) were applied to data meeting these assumptions. When data failed to meet these assumptions, Generalized Linear Mixed Effect Models (GLMM) with appropriate distribution and link functions were used. In cases where models revealed significant effects (p < 0.05), a post hoc Tukey test was performed using the function ‘glht’ from the R package ‘multcomp’. Repeated measurement permutation analysis of variance (PERMANOVA) with ‘Euclidean distance’ matrices and 9999 permutations was used to account for high amount of zeros in our data from *A. rubens*, as this non-parametric test can account for non-normal data, as p-values are calculated by permutations^80^. For PERMANOVA, pair wise tests were used for multiple post hoc comparisons. Details on the applied tests and procedures for all respective response variables are provided in Table S1.

### Ethical approval and informed consent

All methods were carried out in accordance with relevant animal welfare guidelines and regulations.

## Supplementary information

Supplementary information.

## Data Availability

All data are available at PANGAEA: 10.1594/PANGAEA.914600.
