# Supplementary material for: Warming and temperature variability determine the performance of two invertebrate predators
Source: Sci Rep. 2020 Apr 22;10:6780. doi: 10.1038/s41598-020-63679-0 (PMC7176636; doi:10.1038/s41598-020-63679-0)

Supplementary information

Warming and temperature variability determine the performance of two invertebrate predators

List of authors: Sonia C Morón Lugo, Moritz Baumeister, Ola Mohamed Nour, Fabian Wolf, Meike Stumpp and Christian Pansch

Table S1. A factorial design was applied with two fixed factors: mean temperatures (T; ambient vs. warm) and fluctuation regime (F; 'constant' including seasonal temperature shifts vs. sinusoidal fluctuating). Mesocosm identity (M) was included as random factor in all models. In tests addressing *short-term* to *medium-term* variability, individual identity (I) was included as a random factor to account for repeated measurements in the design [79]. PERMANOVA was used to account for high amounts of zeros in our data from *A. rubens*, as this non-parametric test can circumvent the assumption of normality, as p-values are calculated by permutations [80]. Depending on the response variable, Linear Mixed Effect Models (LME), Generalized Linear Mixed Effect Models (GLMM) or Repeated measurement permutation analysis of variance (PERMANOVA) were applied.

	Response variable	Distribution of LME model residuals	Final Model selected	Fixed factors in model	Random factors in model	Test of final model residuals
Asterias rubens	**Overall energy uptake**	not normal and non-homogeneous variance	GLMM (Gamma distribution and logarithm link function)	Te (temperature) x Si (sinusoidal fluctuations)	Me (mesocosm)	Levene´s test (p=0.36)
	**Change in wet weight**	Normal and homogeneous variance	LME	Te x Si	Me	Shapiro test (p=0.14) Levene´s test (p=0.52)
	**Final dry weight**	not normal and non-homogeneous variance	GLMM (Gamma distribution and logarithm link function)	Te x Si	Me	Levene´s test (p=0.44)
	**Energy uptake over *short-term* variability**	not normal with high number of zeros	PERMANOVA ('Euclidean distance' matrices and 9999 permutations)	Te x Ph (phase=time)	Me, In (individual)	-
	**Energy uptake over *medium-term* variability**	not normal with high number of zeros	PERMANOVA ('Euclidean distance' matrices and 9999 permutations)	Te x Si x Pe (period=time)	Me, In	-
Hemigrapsus takanoi	**Overall energy uptake**	Normal and homogeneous variance	LME	Te x Si	Me	Shapiro test (p=0.88) Levene´s test (p=0.20)
	**Change in wet weight**	Normal and homogeneous variance	LME	Te x Si	Me	Shapiro test (p=0.24) Levene´s test (p=0.81)
	**Final dry weight**	Normal and homogeneous variance	LME)	Te x Si	Me	Shapiro test (p=0.83) Levene´s test (p=0.44)
	**Energy uptake over *short-term* variability**	Normal and homogeneous variance	LME	Te x Ph (phase=time)	Me, In	Shapiro test (p=0.35) Levene´s test (p=0.66)
	**Energy uptake over *medium-term* variability**	Normal and homogeneous variance	LME	Te x Si x Pe (period=time)	Me, In	Shapiro test (p=0.05) Levene´s test (p=0.19)

Table S2. Means and 95% CIs of all response variables measured among the four temperature treatments (Ambient, Ambient sinusoidal, Warm and Warm Sinusoidal; A, AS, W, WS, respectively), the phases of the *short-term* sinusoidal fluctuation cycle (Max, Descending, Min and Ascending; Ma, De, Mi, As, respectively) and the *medium-term* periods of temperature variability (Pre-heat, Heat and Post-heat, Pr, He, Po, respectievly) for *Asterias rubens* and *Hemigrapsus takanoi*.

		Asterias rubens				***Hemigrapsus takanoi***			
		**A**	**AS**	**W**	**WS**	**A**	**AS**	**W**	**WS**
**Overall energy uptake (kJ/day)**		0.58 ± 0.24	0.45 ± 0.26	0.08 ± 0.052	0.06 ± 0.024	0.35 ± 0.13	0.31 ± 0.04	0.49 ± 0.08	0.45 ± 0.11
**Change in wet weight (%)**		66.7 ± 45.4	36.5 ± 58.4	-31.8 ± 12.65	-28.8 ± 7.63	101.7 ± 72.51	90.9 ± 71.96	112.48 ± 63.13	63.04 ± 58.33
**Change in wet weight (g)**		10.13 ± 6.69	5.17 ± 8.52	-4.64 ± 1.93	-4.02 ± 1.09	2.32 ± 0.80	2.29 ± 0.80	3.15 ± 0.98	1.79 ± 1.44
**Final dry weight (g)**		4.13 ± 1.05	3.48 ± 1.29	1.77 ± 0.32	1.83 ± 0.18	1.88 ± 0.56	1.79 ± 0.36	2.38 ± 0.57	2.12 ± 0.61
		Ma	De	Mi	As	Ma	De	Mi	As
**Energy uptake over *short-term* variability (kJ/day)**	**AS**	0.06 ± 0.05	0.11 ± 0.07	0.16 ± 0.11	0.12 ± 0.09	0.08 ± 0.01	0.07 ± 0.01	0.07 ± 0.02	0.07 ± 0.01
	**WS**	0	0.11 ± 0.01	0.046 ± 0.02	0.008 ± 0.01	0.11 ± 0.03	0.12 ± 0.02	0.10 ± 0.03	0.07 ± 0.01
		Ph	He	Po		Ph	He	Po	
**Energy uptake over *medium-term* variability (kJ/day)**	**A**	0.16 ± 0.09	0.18 ± 0.09	0.10 ± 0.06		0.06 ± 0.01	0.08 ± 0.04	0.10 ± 0.06	
	**AS**	0.13 ± 0.07	0.10 ± 0.06	0.09 ± 0.07		0.07 ± 0.03	0.07 ± 0.03	0.08 ± 0.04	
	**W**	0.06 ± 0.04	0.003 ± 0.001	0.004 ± 0.009		0.09 ± 0.04	0.12 ± 0.03	0.15 ± 0.03	
	**WS**	0.05 ± 0.02	0	0		0.12 ± 0.05	0.13 ± 0.05	0.10 ± 0.04	

Figure S1. Implemented (line) and realized (squares, circles, and triangles ± SD) temperatures (measured directly within the 2 L experimental units) for the four temperature treatments, ambient, ambient sinusoidal, warm and warm sinusoidal.

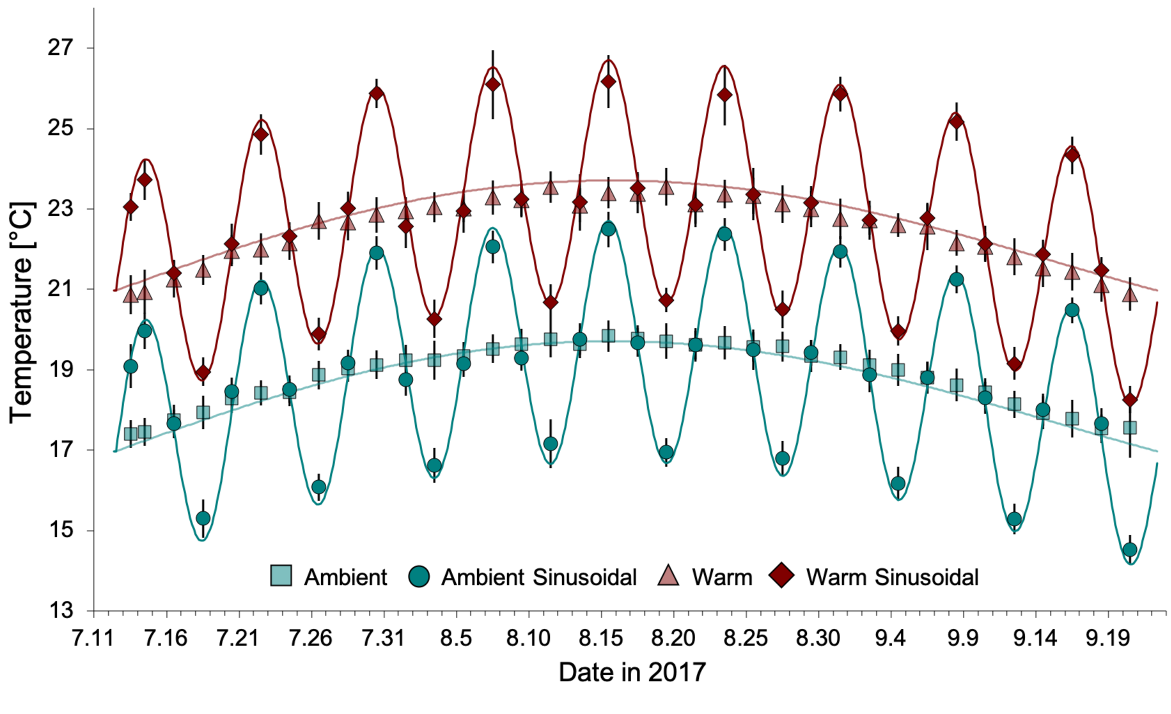

Figure S2. Final dry weight of *Asterias rubens* (**A**) and *Hemigrapsus takanoi* (**B**) during 72 and 64 days of experimentation, respectively, under ambient, ambient sinusoidal, warm and warm sinusoidal temperature treatments. Data are presented as boxplots (median, upper and lower quartile (75th and 25th percentile), whiskers (1.5 times the interquartile range, outliers; N=9). Significant differences between single treatment combinations were tested using a post hoc Tukey test at $p<0.05$ and are indicated by lower-case letters.

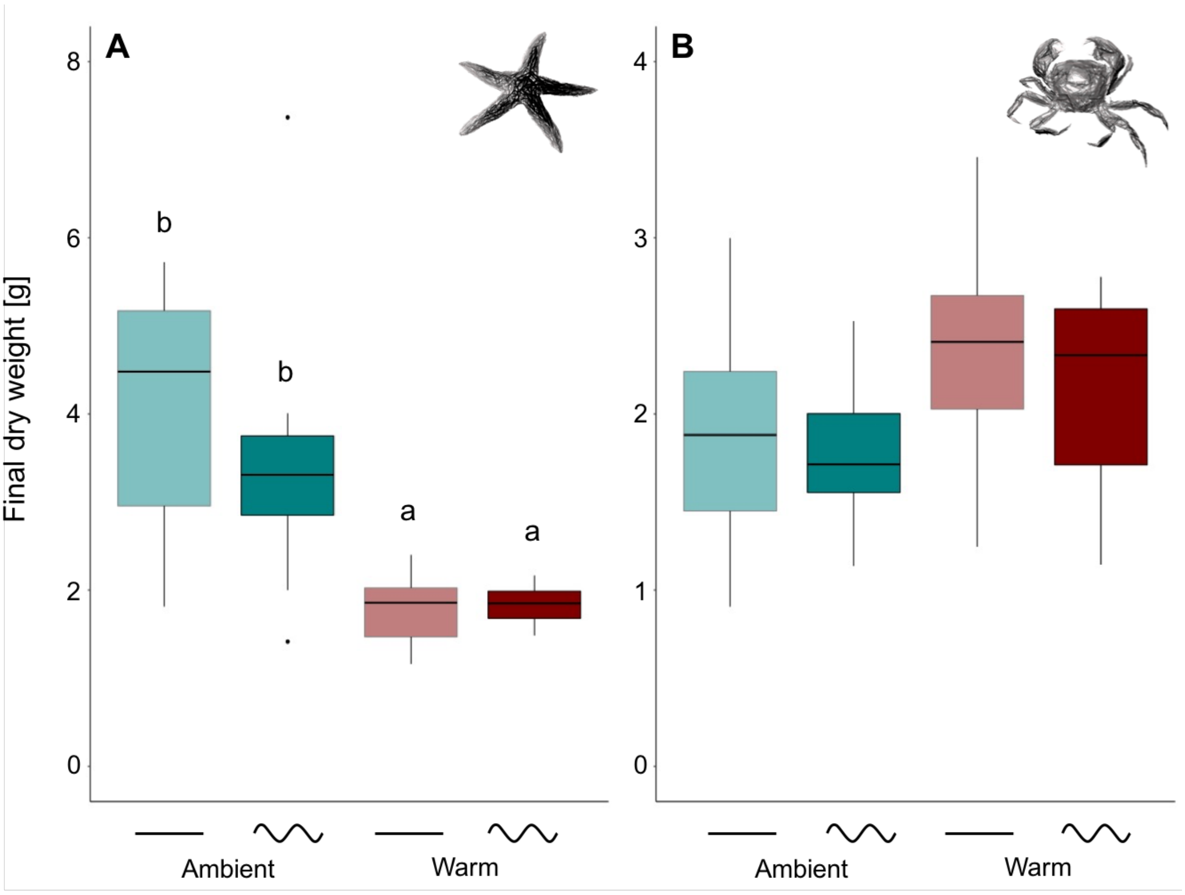

Figure S3. Bi-daily energy uptake of *Asterias rubens* during 72 days of experimentation, under ambient (**A**), ambient sinusoidal (**B**), warm (**C**) and warm sinusoidal (**D**) temperature treatments. Error bars represent standard errors of group means. Underlined are functions of the bi-daily means (‘geom_smooth’ and ‘geom_linerange’ function in ggplot2 in R).

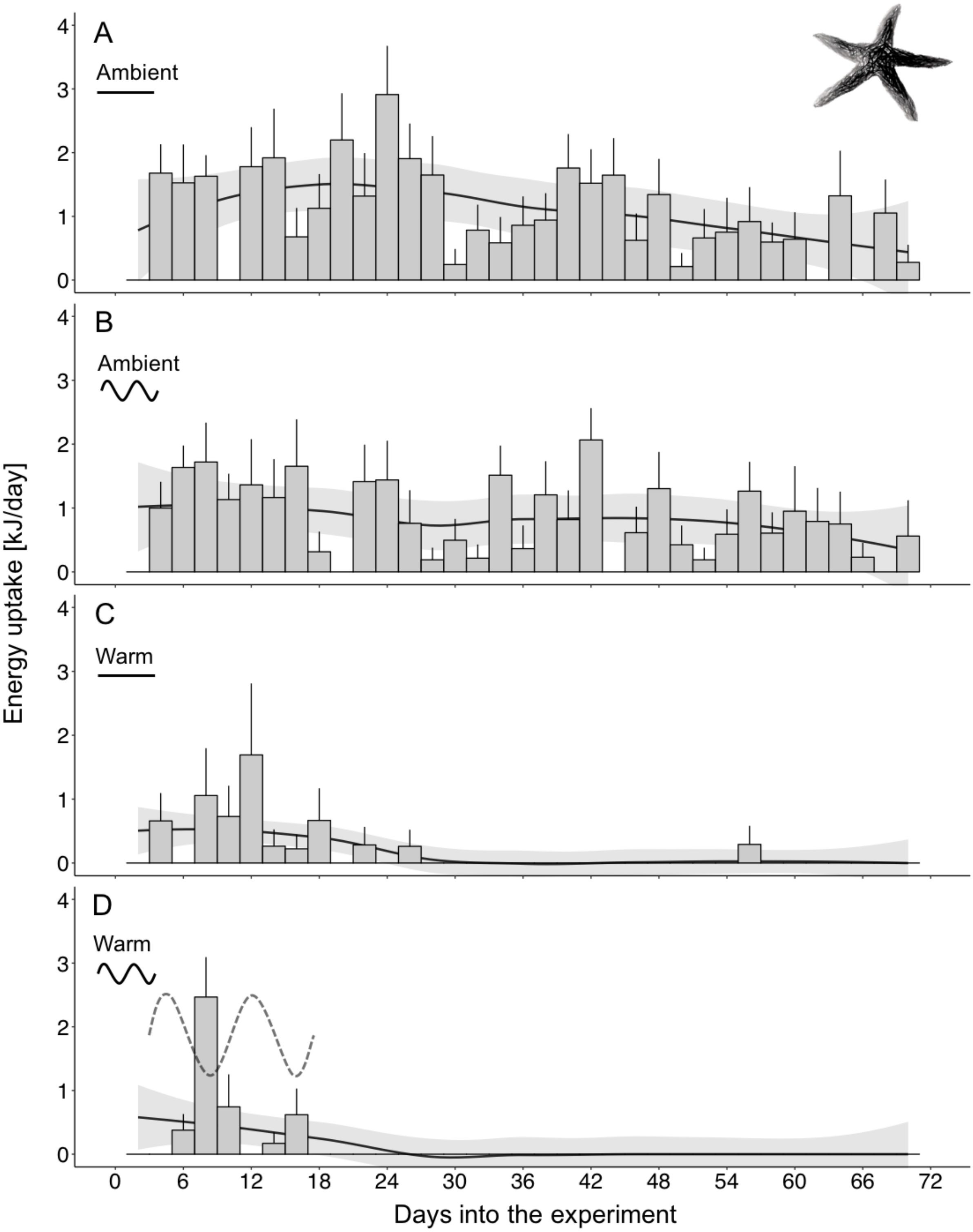

Figure S4. Bi-daily energy uptake of *Hemigrapsus takanoi* during 64 days of experimentation, under ambient (**A**), ambient sinusoidal (**B**), warm (**C**) and warm sinusoidal (**D**) temperature treatments. Error bars represent standard errors of group means. Underlined are functions of the bi-daily means (‘geom_smooth’ and ‘geom_linerange’ function in ggplot2 in R).

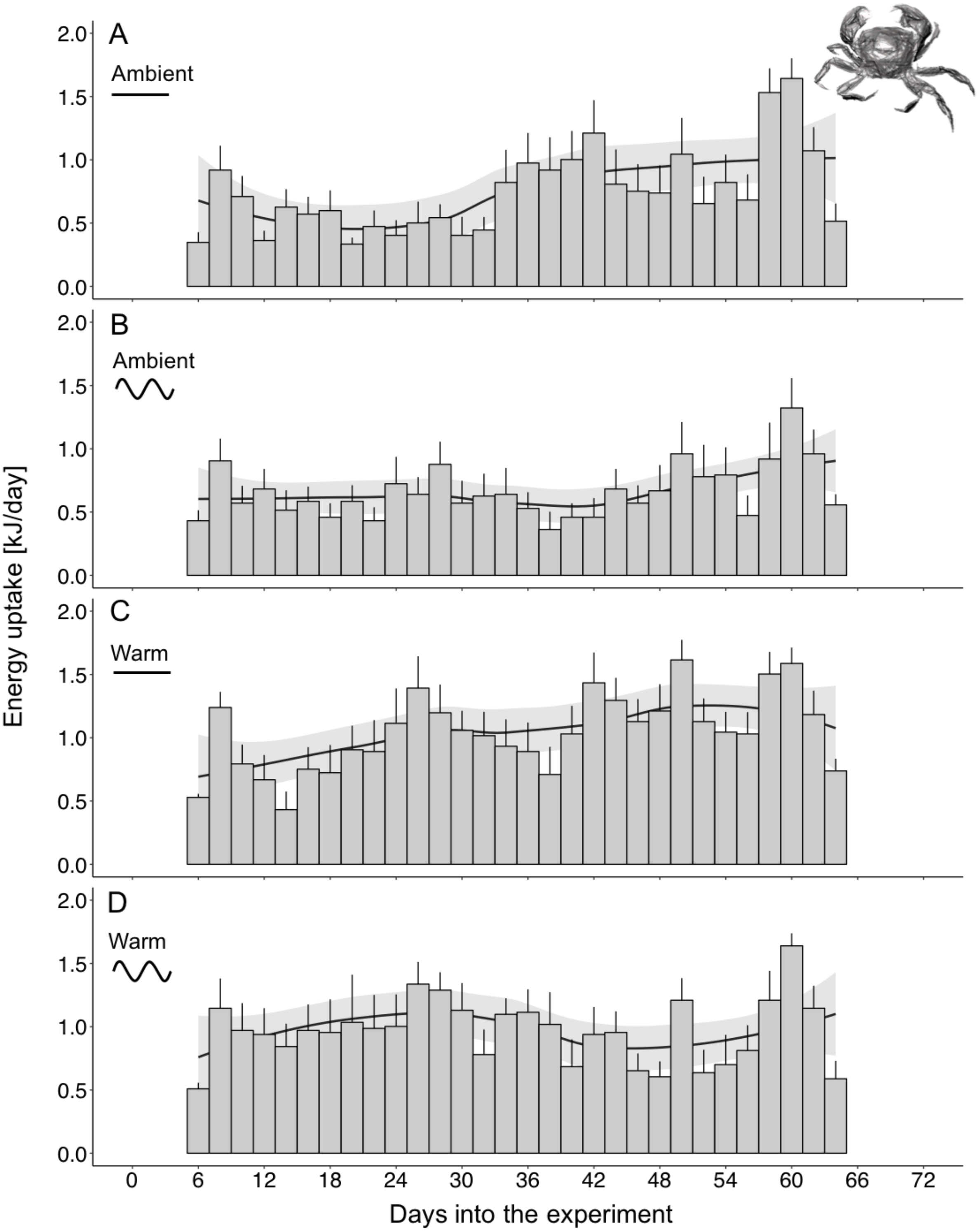

Figure S5. Data for the ambient non-fluctuating treatment (see Fig. 1) were retrieved from surface seawater temperatures from the Kiel Fjord (from 2000 to 2014). The eight warmest years (reaching maximum values above 20°C in summer; coloured lines) were used to retrieve a mean (red line) and its polynomial relationship (fourth degree; dashed grey line), which represents the ambient non-fluctuating temperature treatment in Fig. 1. Data from June to September (experimental period) were considered only (see supplementary in Pansch et al., 2018 for comparisons to the entire dataset). The experimental period is indicated by vertical dashed lines.

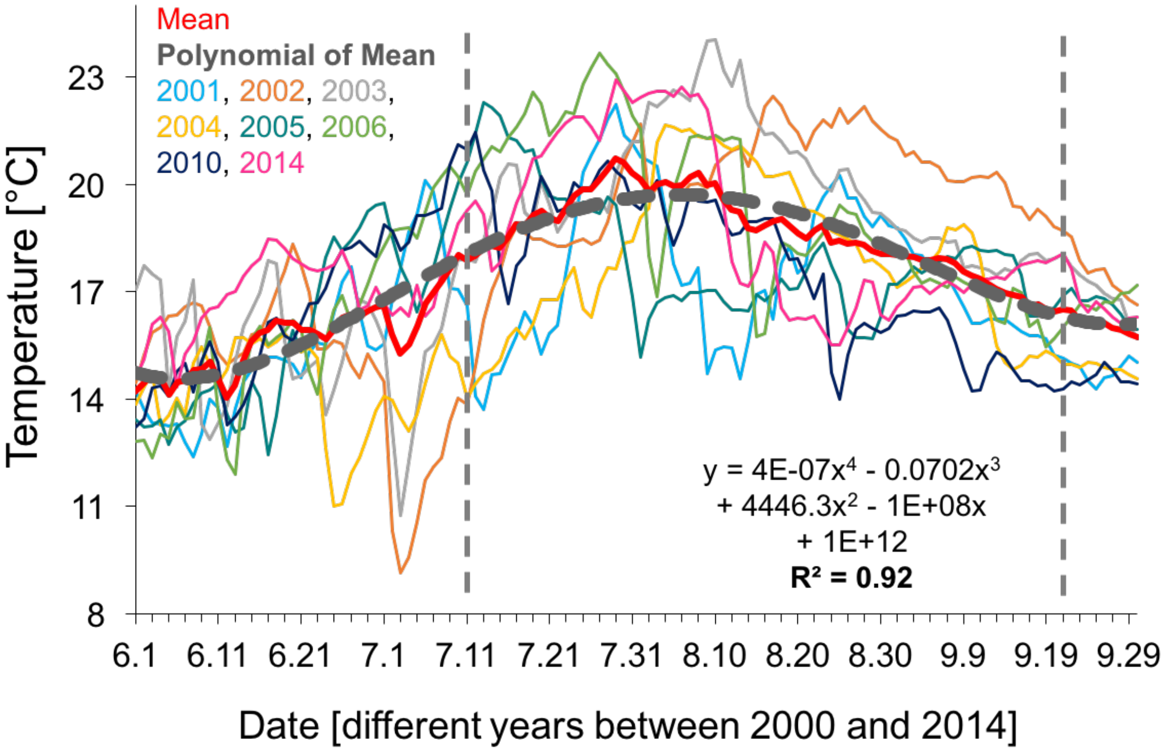

Figure S6. Predictive relationship between shell length and flesh dry weight (soft tissue dry weight) of individuals of *Mytilus edulis* (17.0 to 45.2 mm) from the current experiment as well as data from an existing dataset from the same laboratory for smaller mussels (3.8 to 27.1 mm) from the Kiel Fjord (Thomsen et al. 2013; Pangaea dataset: doi:10.1111/gcb.12109).

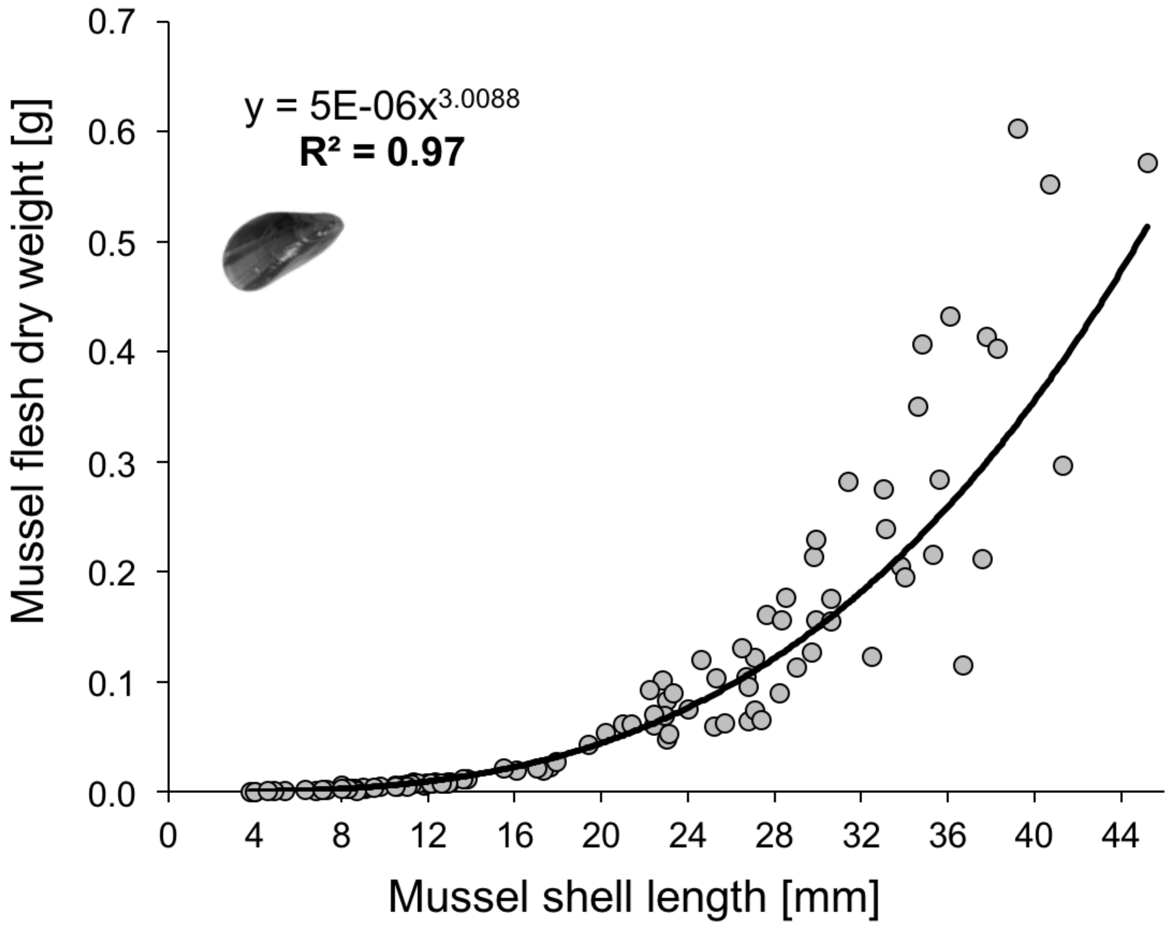

Figure S7. Predictive relationships of dry weight and wet weight (**A** and **B**), size (**C**: maximum length from arm tip to arm tip, **D**: carapace width as distance between the 2 middle antero-lateral teeth) and total area (**D**), of *Asterias rubens* (**A**, **C**, **E**) and *Hemigrapsus takanoi* (**B** and **D**), from the final samplings of the two species from the experiment.

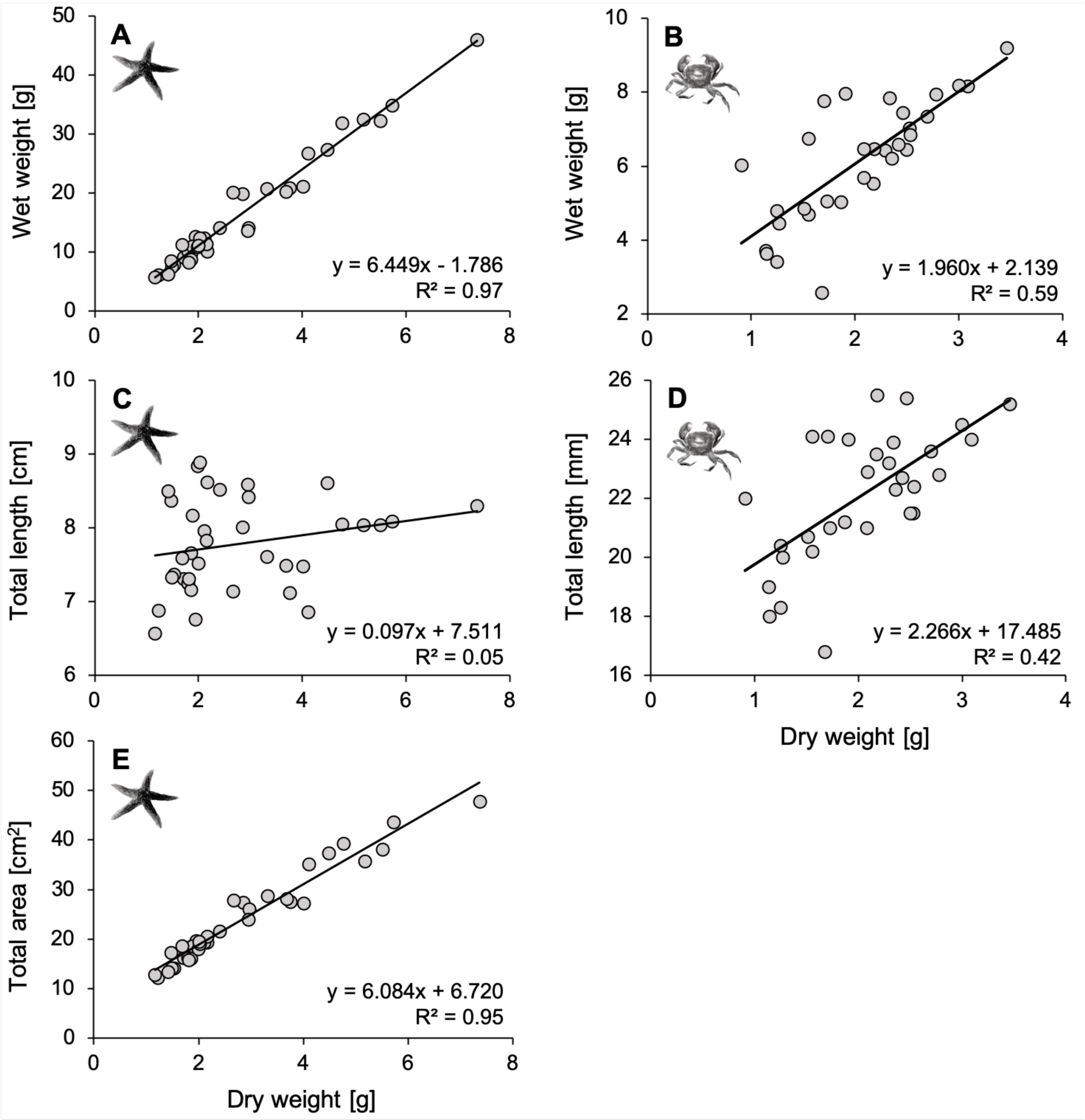

Supplement: Supplementary file 1 — Supplementary information. [file 41598_2020_63679_MOESM1_ESM.pdf]
